# Biventricular repair of double-outlet left ventricle by handmade trileaflet-valved conduit: A case report

**DOI:** 10.1097/MD.0000000000032070

**Published:** 2022-11-25

**Authors:** Ailixiati Alifu, Haifan Wang, Yuntian Su, Renwei Chen

**Affiliations:** a Department of Cardiothoracic Surgery, Hainan Women and Children’s Medical Center, Haikou, Hainan Province, China.

**Keywords:** biventricular repair, case report, congenital cardiac malformation, double outlet left ventricle, handmade conduit

## Abstract

Double-outlet left ventricle (DOLV) is a rare congenital cardiac malformation in which both great arteries arise entirely or predominantly from the left ventricle. An extracardiac conduit is the first surgical option for repairing DOLV, specifically because its placement of the extracardiac conduit can be customized to accommodate all possible anatomical variations. The bovine jugular veins and homograft valves are often used as conduits. There have been no reports on the use of handmade trileaflet-valved conduits for correcting DORV.

**Patient concerns:**

A 1-year old male was admitted for significant heart murmur and cyanosis, according to the results of transthoracic echocardiography, computed tomography angiography, and cardioangiography, and was diagnosed with DOLV and pulmonary stenosis.

**Diagnosis and interventions:**

The patient underwent biventricular repair with a handmade trileaflet-valved extracardiac conduit. The postoperative course was uneventful.

**Outcome:**

Three months after the surgery, TTF indicated mild right ventricular outflow obstruction and pulmonary valve regurgitation.

**Lessons:**

Correction of the left ventricular double outlet with a handmade trileaflet-valved conduit has been shown to have excellent performance, and long-term outcomes should be followed over time.

## 1. Introduction

Double-outlet left ventricle (DOLV) is a type of ventriculoarterial connection in which both great arteries arise entirely or predominantly from the morphological left ventricle.^[1]^ It is associated with a misaligned ventricular septal defect (VSD), various degrees of hypoplasia of the right ventricle, and the presence or absence of pulmonary stenosis.^[1]^ This malformation occurs in <1 in 200,000 births.^[2]^ Anomaly of conus development (either unilaterally absent or bilaterally absent) and anomalous absorption or misorientation of the junction of both major arteries of the right ventricle in the subarterial segment of the ventricular septum are the 2 main embryogenic reasons for this phenomenon.^[3]^ Repair of the double outlet of the left ventricle using an extracardiac conduit, bovine jugular veins, or homograft valves is commonly performed. In our case, we used a handmade trileaflet-valved conduit and achieved good outcomes.

## 2. Case presentation

**Figure 1.**
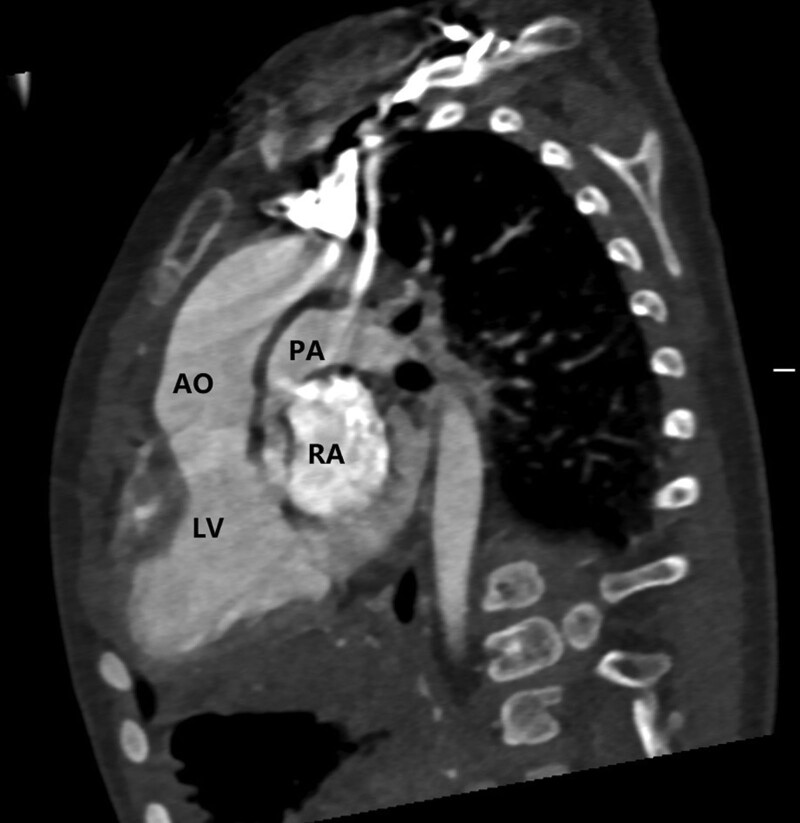
Computed tomography angiography (CTA) shows that the aorta located anterior to the pulmonary artery which was stenosis.

**Figure 2.**
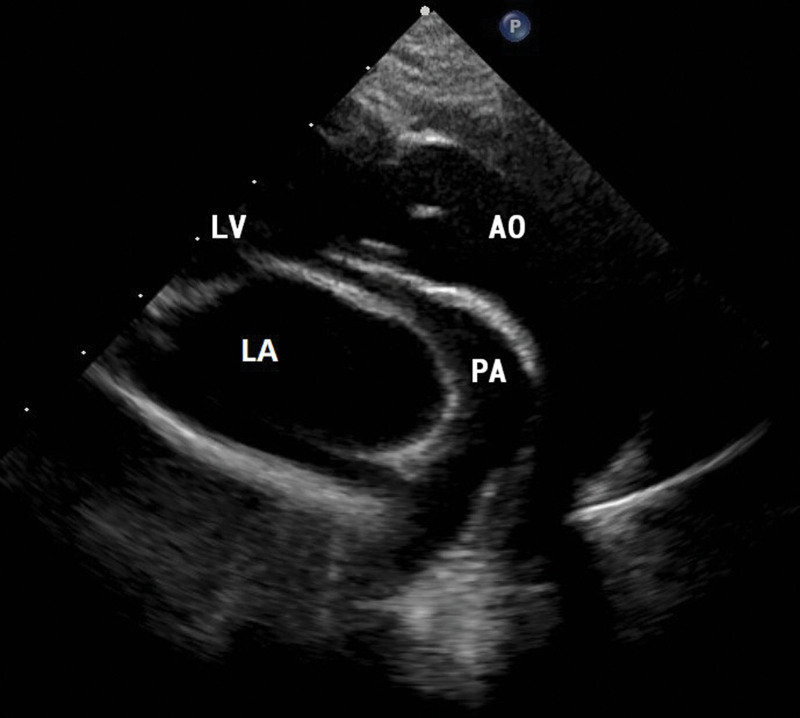
Transthoracic echocardiography (TTE) shows that the both great artery originated from LV, LV = left ventricle.

**Figure 3.**
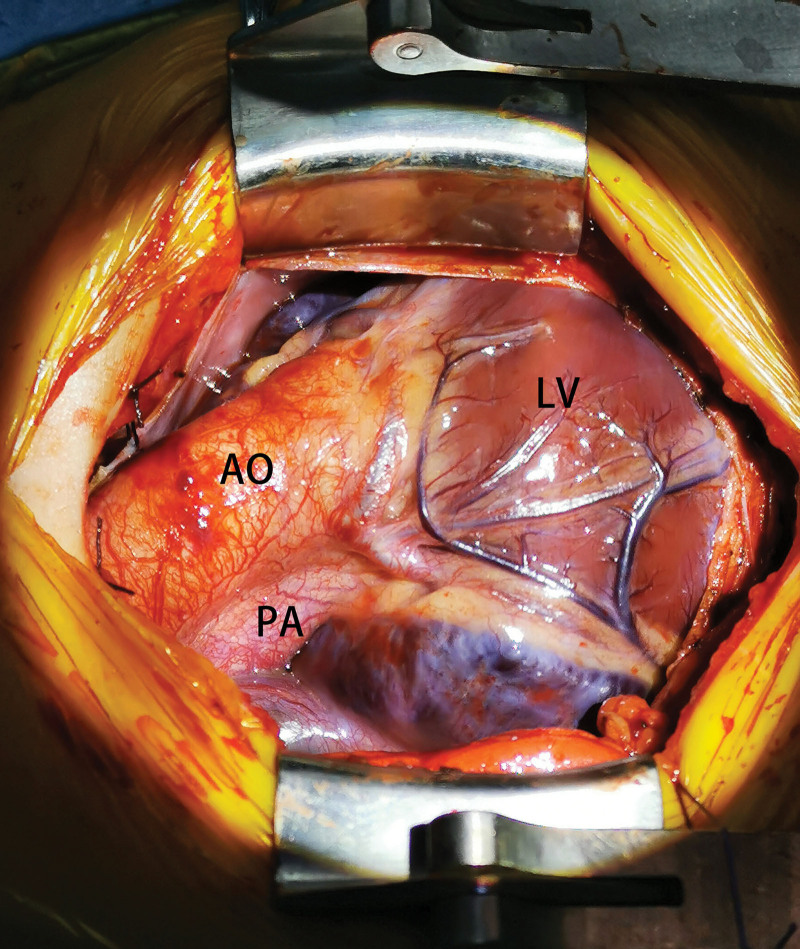
Operation view: aorta located right front of the pulmonary artery.

**Figure 4.**
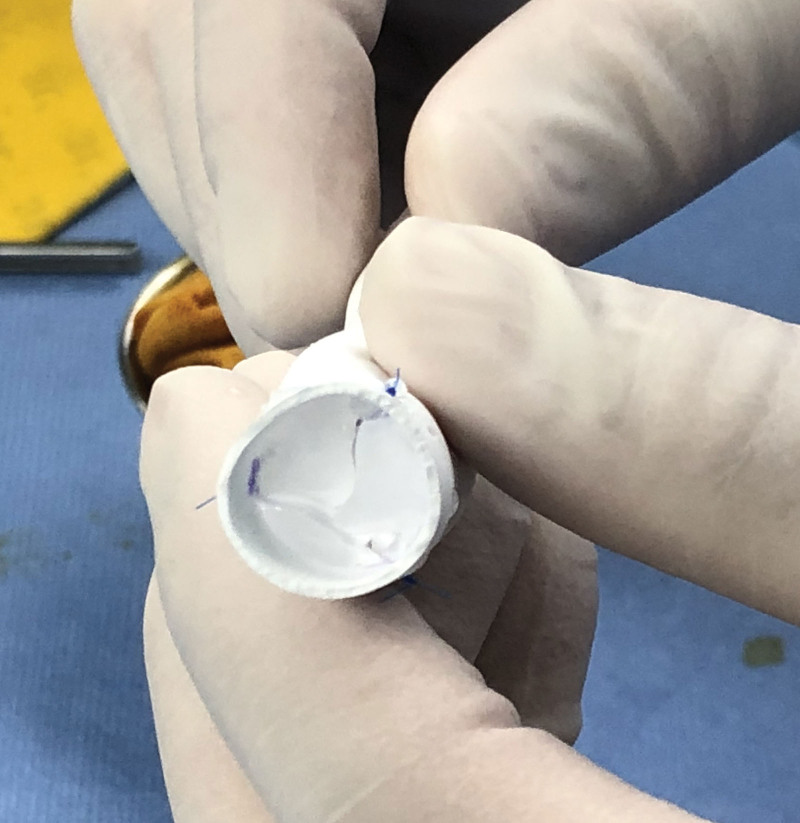
Hand-made trileaflet-valved conduit.

**Figure 5.**
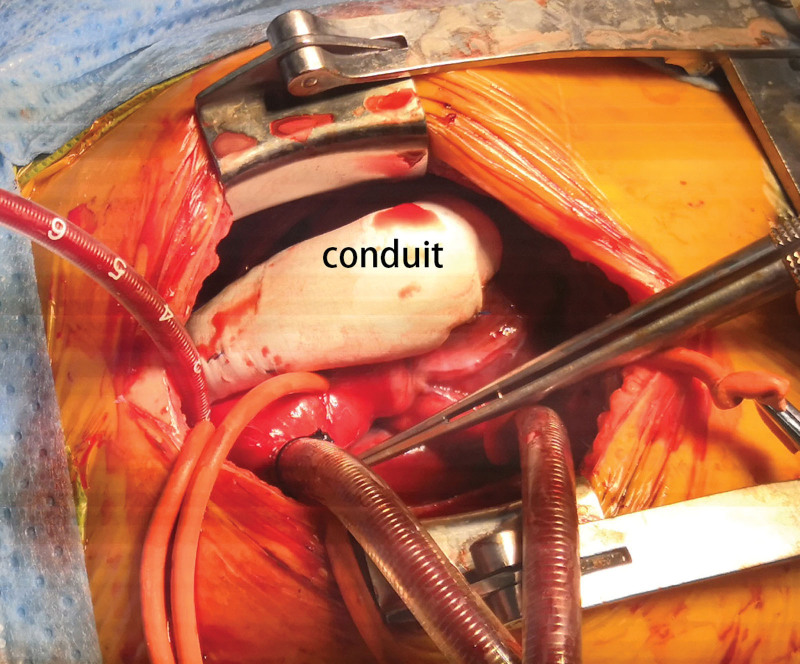
Implanted conduit.

**Figure 6.**
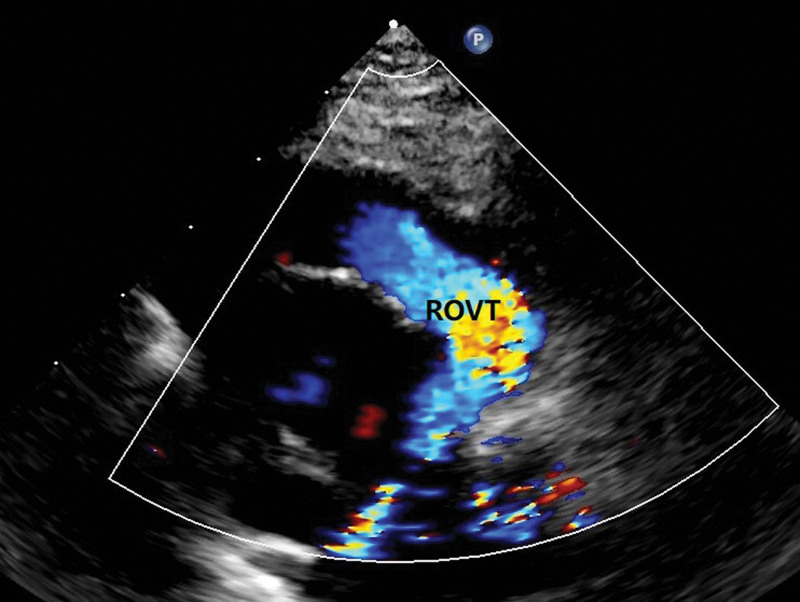
The transthoracic echocardiography (TTE) follows up at 3 months.

A 1-year-old male was admitted to our department with a significant heart murmur and moderate cyanosis, without a family history of cardiac disease. Physical examination revealed a skeletal figure, cyanosis, and a systolic heart murmur over the left intercostal space. Computed tomography angiography (Fig. 1) and transthoracic echocardiography (TTE) (Fig. 2) examinations showed that the heart had levocardia, situs solitus, and concordant atrioventricular connections. The aorta was positioned to the left and anterior to the pulmonary trunk, mainly arising from the morphological left ventricle. There was a 10 × 9 mm nonrestrictive malalignment VSD below the aortic valve. The tricuspid valve diameter was 12 mm in TTE apical 4-chamber views, and the Z-score was −1.53. Pulmonary artery stenosis, secundum atrial septal defect, patent ductus arteriosus (PDA), and right lateral aortic arch were also detected. Electrocardiography revealed right ventricular hypertrophy. Chest radiography revealed enlargement of the heart. Laboratory examination revealed a blood hemoglobin level of 194 g/L. Subsequently, the patient was diagnosed with DOLV and pulmonary artery stenosis, which was confirmed via cardiac catheterization. The patient underwent standard median sternotomy. On extracardiac inspection, the aorta was in front of the pulmonary artery with a right lateral aortic arch. There was stenosis of both the pulmonary trunk and left pulmonary artery (Fig. 3). The left anterior descending and circumflex coronary arteries arise as a single trunk from Sinus 1 and the right coronary artery from Sinus 2. CPB was initiated after cannulation of the ascending aorta, right atrium, and the right inferior vena cava. The patent ductus arteriosus was then double ligated and dissected. The aorta was cross-clamped with antegrade cardioplegic arrest, and intracardiac findings confirmed that the VSD was located just below the aortic valve, which is completely located on the morphologic left ventricle, and the main part of the pulmonary valve did so. The anterior wall of the right ventricular outflow tract was incised, and the VSD was closed with continuous sutures using a pericardial patch. The pulmonary trunk was incised just above the root and the proximal portion was closed with horizontal mattress sutures. Simultaneously, an assistant accomplished the extracardiac conduit, as shown in the process.^[4]^ The circumference of the 18 mm Gore-Tex tube was marked at 3 equal points after the inner surface was turned inside out. A 0.1 mm polytetrafluoroethylene sheet was cut into 3 equally connected U-shaped units based on the mold. Use a 5-0 prolene suture line was used to interrupt the U-row connecting part with 3 equal points on the tube, and continuously sew along the U-shaped edge. Subsequently, a 3-valve structure is formed (Fig. 4). After tube restoration, a single stitch was intermittently sutured at the root junction of the 3 valves to complete the valved conduit. The bifurcation of the left and right pulmonary arteries was anastomosed distally proximal to the right ventriculostomy site (Fig. 5). Postoperative intensive care was uneventful, and mechanical ventilation was ceased on the 4th postoperative day of postoperation. We prescribed warfarin as an oral anticoagulant, and the INR was maintained at 1.8 to 2.5 seconds. The child was discharged on the 10th day of postoperative. TTE (Fig. 6) follow-up at 3 months was excellent.

## 3. Discussion

DOLV is an anatomically and surgically challenging subset in which the main arteries originate predominantly or entirely from the morphological left ventricle. There are multiple options for biventricular repair based on the anatomy of the great arteries, location of the VSD, and the presence of pulmonary stenosis.^[1]^ Sakakibara et al reported complete intracardiac repair in 1967 using patch intraventricular tunnel repair to connect the VSD to the pulmonary semilunar valve.^[5]^ Kerr et al reported a case in 1971 in which right ventricular-to-pulmonary artery continuity was maintained by forming a roof surrounding the ventriculostomy and pulmonary arteriotomy using a pericardial patch.^[6]^ In 1973, Pacifico et al described reconstruction using a valved conduit linking the right ventricle to the main pulmonary artery.^[7]^ Chiavarelli et al reported the arterial repair of DOLV in 1992 by utilizing pulmonary root translocation into the right ventricle, including the intact pulmonary valve.^[8]^ Sharratt et al were the first to describe Fontan-type repair of DOLV with a hypoplastic right ventricle.^[9]^ Conventionally, Rastelli-type repair is performed with interposition of the right ventricle to the main pulmonary artery conduit in patients with severe pulmonary stenosis or atresia. The versatility of extracardiac conduit insertion remains the primary option for repairing DOLV.^[10]^ All reports on external conduits used to repair DOLV used bovine jugular vein conduits and pulmonary homografts. Trileaflet-valved conduits are typically used to repair malformations of the right ventricular outflow tract,^[11]^ and there have been no reports on their use in fixing LVOT malformations. This conduit was chosen because it seems to be associated with lower rates of postoperative complications, graft failure, and early phase mortality (especially in younger patients) than bovine jugular vein conduits.^[11,12]^ We prescribed warfarin for oral anticoagulation, and the INR was maintained at 1.8 to 2.5. Three months after the surgery, TTF indicated mild right ventricular outflow tract obstruction and pulmonary valve regurgitation.

## 4. Conclusion

In the present study, we report a case of DOLV, a rare congenital heart disease that was treated using a handmade trileaflet-valved conduit, which is not the first option for correcting such malformations. The major limitation of extracardiac repair is conduit-related morbidity, particularly the need for re-intervention. The child requires long-term follow-up to determine if it needs to be replaced or if there are signs of failure.

## Acknowledgments

We formally acknowledge the patient and her family for entrusting our team with the clinical care.

## Author contributions

**Conceptualization:** Ailixiati Alifu, Yuntian Su.

**Data curation:** Haifan Wang.

**Writing – original draft:** Ailixiati Alifu.

**Writing – review & editing:** Ailixiati Alifu, Renwei Chen.
